# CLIMATE CHANGE AND LONGEVITY IN EUROPE: A SCOPING REVIEW ON HEALTH CARE USE DURING EXTREME AIR TEMPERATURES

**DOI:** 10.1093/geroni/igae098.1008

**Published:** 2024-12-31

**Authors:** Linda Enroth, Laura Kananen, Ella Tapola, Jutta Pulkki

**Affiliations:** Tampere University, Finland, Tampere, Pirkanmaa, Finland; SOC/Tampere University, Finland, Tampere, Pirkanmaa, Finland; Tampere University, Tampere, Pirkanmaa, Finland; Tampere University, Tampere, Pirkanmaa, Finland

## Abstract

Climate change together with increasing longevity pose challenges for social, economic, and ecological sustainability of the health care systems. The associations of hot and cold temperatures with higher mortality are well understood, but less is known of their impacts on health care utilization. This scoping review examines how extreme air temperatures are associated with healthcare use in 65+ populations in Europe. We screened peer-reviewed articles from six databases (Medline, Science Direct, Web of Science, Scopus, ProQuest and Greenline) published in English 2000-2022. The key search terms were air temperature, older people, health care services, and Europe. We screened 1,457 articles, reviewed 121 full-texts, and extracted data from 43 articles. Half of the articles examined heat exposure, ten cold exposure, and 12 examined both. Emergency hospital admission was the most commonly examined health service. 20 out of 33 (heat) and 16 out of 22 (cold) studies found a positive association between extreme air temperature and health care use. Most studies were patient-based and the most common conditions leading to health care use were cardiovascular and respiratory diseases. Only three studies investigated the modifying impact of social vulnerabilities. They found a tendency that social and material deprivation contributes to a higher risk of health care use during extreme temperatures. Research on health care use under extreme temperatures varies in quality and mostly reflects exacerbation of underlying chronic conditions. There is a need for further research that uses large data sets, considers multidimensional modifying risk factors, and reports results separately for older people.

